# The landscape of NUP98 rearrangements clinical characteristics and treatment response from 1491 acute leukemia patients

**DOI:** 10.1038/s41408-024-01066-y

**Published:** 2024-05-14

**Authors:** Jie Tian, Yongmei Zhu, Jianfeng Li, Guang Yang, Xiangqin Weng, Ting Huang, Lingling Zhao, Haimin Sun, Zeying Yan, Sujiang Zhang

**Affiliations:** 1 Department of Hematology, Ruijin Hospital, Shanghai Jiao Tong University School of Medicine, Ruijin Road II 197, Shanghai, China; 2 National Research Center for Translational Medicine at Shanghai, Shanghai Institute of Hematology, State Key Laboratory of Medical Genomics, Ruijin Hospital, Shanghai Jiao Tong University School of Medicine, Ruijin Road II 197, Shanghai, China

**Keywords:** Acute myeloid leukaemia, Acute myeloid leukaemia

Dear Editor,

Nucleoporin 98 (NUP98, chromosome 11p15) fusion oncoproteins also called NUP98 rearrangements (NUP98r) have already been identified in a spectrum of hematologic malignancies for a long time, including acute myeloid leukemia (AML), chronic myeloid leukemia in blast crisis/accelerated phase, chronic myelomonocytic leukemia, myelodysplastic syndrome, acute lymphoblastic leukemia (ALL) especially T-ALL and mixed-phenotype acute leukemia, mostly associated with pediatric leukemias and poor prognosis [1–4]. NUP98r generate NUP98 fusion proteins that join the N-terminal domain of NUP98 with various C-terminal partners including HOX genes and non-HOX genes [1]. In 2022 ELN classification, NUP98r especially including NUP98::NSD1, NUP98::KDM5A and other partners were identified as AML with other rare recurring translocations [5]. In 2022 WHO classification, three AML types with characteristic rearrangements involving lysine methyltransferase 2A (KMT2A), MECOM and NUP98 were recognized [6]. It was also important to note that rearrangements involving these three genes, particularly NUP98, may be cryptic on conventional karyotyping. Although a comprehensive report about pediatric NUP98r leukemia patients has just been published recently [7], there was no overall report about adult NUP98r leukemia patients.

In our previous study, we have demonstrated that NUP98::NSD1 positive AML showing initially poor treatment response can benefit from FLT3 inhibitors (FLT3i) and venetoclax (VEN) as well as hematopoietic stem cell transplantation (HSCT), suggesting the important treatment role of FLT3i or/and VEN therapy and HSCT in this subgroup patients [8]. Recent studies also indicated that NUP98r in leukemogenesis may be associated with their interaction of menin with KMT2A [9]. Clinical trials have also shown the efficacy of menin inhibitors in the treatment of NUP98r leukemia patients, and further studies also demonstrated that acquired resistance to menin inhibitors may be attributed to somatic mutations in MEN1 gene [10]. Here, we reported a large cohort of NUP98r leukemia patients from our center, drawing a landscape of adult NUP98r leukemia, suggesting the available treatment including FLT3i, VEN and HSCT may remarkably improve the poor prognosis, especially for NUP98::NSD1 positive leukemia patients.

A total of 55 NUP98r positive leukemia patients from 1491 acute leukemia (AL) patients (3.7%, 55/1491), individually including 51 of 1099 AML (4.6%, 51/1099) and 4 of 392 ALL (B-ALL and T-ALL, 1.0%, 4/392), were identified by RNA sequencing (RNA-seq) in our center from May 2019 to May 2023, including 28 female and 27 male, with a median age of 40 years (4–79 years). These patients were mostly newly diagnosed AL according to bone marrow morphology and immunology including 51 AML which were further divided into de novo AML (45/51), therapy-related AML (t-AML, 3/51) and secondary AML (s-AML, 3/51), 3 early T-cell precursor ALL (ETP-ALL), and 1 B-ALL. Furthermore, the proportion of NUP98r in de novo AML, t-AML and s-AML was 4.3% (45/1039), 7.1% (3/42) and 16.7% (3/18), respectively, also suggesting higher NUP98r ratio in s-AML (Table S1).

The median white blood cell counts of these patients was 61.1 × 10^9^/L (1.5–369 × 10^9^/L) and platelet counts were 61 × 10^9^/L (5–267 × 10^9^/L) individually (Tables S1 and S2). Twenty-seven NUP98r patients were identified as WBC > 50 × 10^9^/L at the first diagnosis (27/55, 49.1%), including 17 NUP98::NSD1 patients (17/27, 63.0%), and 23 patients with FLT3 mutations (23/27, 85.2%). Similarly, 16 patients were identified as WBC > 100 × 10^9^/L at the first diagnosis (16/55, 29.1%), including 12 NUP98::NSD1 patients (12/16, 75.0%), and 13 with FLT3 mutations (13/16, 81.3%), suggesting for WBC > 50 × 10^9^/L in clinical practice we should pay close attention to checking NUP98r especially NUP98::NSD1.

In our study, 3 NUP98r AML patients (3/51, 5.9%) were <18 years of age with all showing NUP98::NSD1, and 48 NUP98r AML patients (48/51, 94.1%) were >18 years of age, including 22 NUP98::NSD1 (45.8%, 22/48) and 10 NUP98::HOXA9 (20.8%, 10/48). Similarly, 10 NUP98r AML patients (10/51, 19.6%) were >60 years of age with 5 NUP98::NSD1 (50%, 5/10), and 41 patients (41/51, 80.4%) were <60 years of age with 20 NUP98::NSD1 (48.8%, 20/41) and 9 NUP98::HOXA9 (22.0%, 9/41).

Cytogenetic results were available from 53 patients, showing 24 patients harboring normal karyotypes and 29 patients harboring various cytogenetic abnormalities including characteristic chromosome translocations involving NUP98 (Table S1).

In our patient group, a total of 15 different NUP98 fusions were identified. The most prevalent NUP98 fusion was NUP98::NSD1, which was the same as pediatric patients [7] and totally identified in 25 AML patients by RNA-seq (Table S1). However, no characteristic *t* (5; 11) (q35.3; p15.4) was found in these patients, which may be related to the fact that NUP98 and NSD1 genes are located at the end of chromosomes and not easily detected by conventional karyotype analysis. The second fusion was NUP98::HOXA9, which was different from pediatric patients (NUP98::KDM5A) [7]. NUP98::HOXA9 fusion was totally identified in 10 AML patients, and cytogenetic results showed that characteristic *t* (7; 11) (p15; p15) can be identified in almost NUP98::HOXA9 positive patients except one patient result was not available (Table S1). The third NUP98 fusion was NUP98::PRRX2 which was identified in 4 AML patients. NUP98::HMGB3, NUP98::TOP1 and NUP98::KDM5A were all identified in 2 AML patients individually and NUP98::CCDC28A was also identified in 2 patients but both were ETP-ALL. Finally, NUP98::TNRC18, NUP98::HOXA11, NUP98::HHEX, NUP98::DDX10, NUP98::PSIP1 and NUP98::KMT2A were all identified in only one AML patient, and NUP98::LNP1 was identified in only one ETP-ALL patient. It should be mentioned that NUP98::ASNSP4 as an unreported new fusion was just identified in a B-ALL patient (Table S1). It should also be mentioned that the partner gene of NUP98 in de novo AML was NSD1 (24), HOXA9 (9), PRRX2 (3), HMGB3 (2) and others (7), whereas in t-AML were NSD1 (1), HOXA9 (1) and TOP1 (1), and in s-AML were PRRX2 (1), KDM5A (1) and DDX10 (1) individually.

**Fig. 1:**
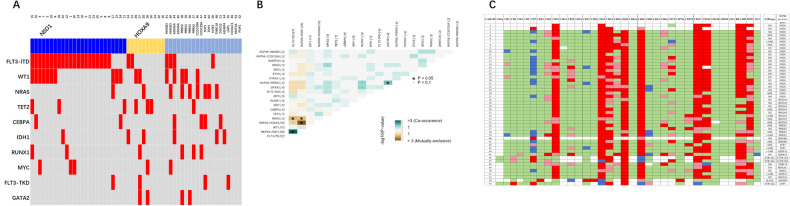
The clinical characteristics of NUP98 leukemia patients. **A** Relationship between NUP98 fusions and concurrent mutations. Top 10 mutations were shown. **B** Co-occurrence and mutual exclusion analysis. Only top 20 mutation and NUP98 fusions were shown. Both (**A**) and (**B**) demonstrated NUP98::NSD1 and FLT3-ITD were the mostly concurrent gene abnormalities. **C** Heatmap depicting the immunophenotypic landscape of patients. Positive markers were shown with red, partial positives were shown with pink, cross-lineage antigen positives were shown with blue and negative markers were shown with green.

As for gene mutations in these NUP98r patients (Figs. S1 and S2), FLT3-ITD was the most common concomitant mutation, and undoubtedly it was closely associated with NUP98::NSD1 (21/25, 84%) (Fig. 1A, B). However, it was also associated with other NUP98r including 2 NUP98::HOXA9 (2/10, 20%), 2 NUP98::HMGB3 (2/2, 100%), 1 NUP98::KDM5A (1/2, 50%) and 1 NUP98::HHEX (1/1, 100%) (Fig. 1A). The second concomitant mutation was WT1 mutation, which was also associated with NUP98::NSD1 (40%, 10/25), and other NUP98r including 2 NUP98::HOXA9 (2/10, 20%), 2 NUP98::PRRX2 (2/4, 50%), 1 NUP98::HMGB3 (1/2, 50%), 1 NUP98::KDM5A (1/2, 50%) and 1 NUP98::CCDC28A (1/2, 50%) (Fig. 1A).

As for immunophenotype, NUP98::NSD1 positive AML characteristically demonstrated CD105 negative, CD36 partial positive and CD123 positive, suggesting CD34st+CD117st+HLA-DRst+CD38st+CD33st+CD13dim+CD105−CD36p+CD123+ may be common immunophenotype of this subgroup. In addition, even though most of NUP98 fusions in our patients group were associated with AML immunophenotype, a few were found to be associated with ETP-ALL including 2 NUP98::CCDC28A and 1 NUP98::LNP1, and 1 NUP98::ASNSP4 associated with B-ALL immunophenotype (Fig. 1C).

Moreover, NUP98::CCDC28A was found in ETP-ALL, with cytogenetics showing chromosome 6q aberrations, suggesting its potential role in leukemogenesis of ETP-ALL which has been previously reported in AML and T-ALL [1, 11]. NUP98::LNP1, which has been reported in AML [1], was also identified in ETP-ALL in our patients, with cytogenetics showing *t* (3; 11) (q12; p15), also suggesting its potential role in leukemogenesis of ETP-ALL (Table S1). NUP98::ASNSP4, as the only identified NUP98 fusion in B-ALL in our patient series, with normal karyotype, and ASNSP4 as a pseudogene, was awaiting clarification of this fusion’s biological role in B-ALL. There was no any other report about NUP98r and B-ALL except Liu’s report [12]. However, the partner gene of NUP98 in this study was not identified only showing hyperdiploidy.

**Fig. 2:**
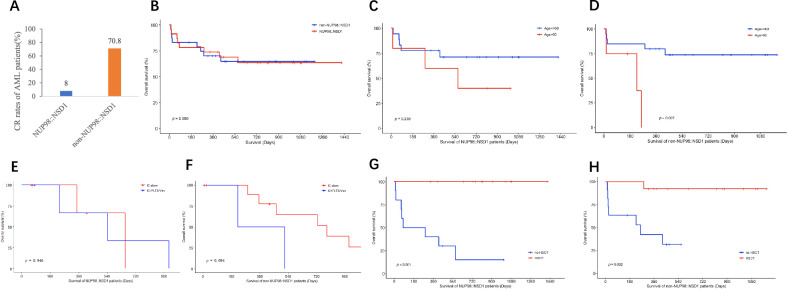
The analysis of CR rate and OS in NUP98::NSD1 and non-NUP98::NSD1 AML patients. **A** CR rate of NUP98::NSD1 and non-NUP98::NSD1 patients in AML. Kaplan–Meier curve of OS based on (**B**) NUP98::NSD1 and non-NUP98::NSD1 in 51 AML patients, (**C**) age of NUP98::NSD1 AML patients, (**D**) age of non-NUP98::NSD1 AML patients, (**E**) induction and salvage therapy of NUP98::NSD1 AML patients younger than 60 years, (**F**) induction and salvage therapy of non-NUP98::NSD1 AML patients younger than 60 years, (**G**) HSCT of NUP98::NSD1 AML patients, (**H**) HSCT of non-NUP98::NSD1 AML patients.

In terms of initial induction treatment in 51 AML patients, for NUP98::NSD1, 20 patients received intensive induction chemotherapy (IC), 4 received low-intensity therapy (LIT) with VEN and 1 received IC with FLT3i. For non-NUP98::NSD1, 16 patients received IC, 4 received LIT + VEN, 4 received LIT alone, 1 received IC with FLT3i and 1 received IC with VEN. The complete remission (CR) rate was 37.3% (19/51) in the total AML patients, NUP98::NSD1 patients showed a significantly lower frustrating CR rate of 8.0% (2/25) and non-NUP98::NSD1 patients showed 70.8% (17/24) (*p* < 0.001) individually (Fig. 2A), which was also in line with the previous report [13]. However, the CR rates of NUP98::NSD1 patients who received LIT + VEN treatment were 50% (2/4), showing strikingly advantage comparing with IC alone (0%, 0/20) (*p* = 0.024), suggesting the depressed treatment outcome of IC alone and the potential important role of VEN-based regimen in the induction therapy of NUP98::NSD1 patients. Only a few studies mentioned the poor response to traditional IC regimen and the benefit of VEN and/or FLT3i introduction in NUP98::NSD1 AML patients [14, 15]. Other patients who received IC + FLT3i showed partial remission. In non-NUP98::NSD1 patients cohort, the CR rate of patients who received LIT + VEN was 100% (4/4) comparing with IC alone (64.3%, 9/14) (*p* = 0.278). In addition, 1 non-NUP98::NSD1 patient received IC + VEN and 1 received IC + FLT3i both achieved CR, and 2 of 4 LIT alone achieved CR (2/4, 50%).

In 51 AML patients, after the median 30 months follow-up (range, 0.4~47.1 months), including 24.6 months (range, 0.4~47.1 months) for NUP98::NSD1 patients and 32.2 months (range, 0.5~39.0 months) for non-NUP98::NSD1 patients, the overall survival (OS) data of 47 patients were available. The 4-year OS of NUP98::NSD1 patients (65.2%, 15/23) was similar to non-NUP98::NSD1 (66.7%, 16/24) (*p* = 0.950) (Fig. 2B), and this comparability was remarkable different from the wide CR rate gap between these two patients group, maybe attributed to the salvage regimen especially containing VEN and FLT3i as well as subsequent HSCT. Further analysis demonstrated a total of 16 NUP98::NSD1 patients received salvage therapy after intensive induction therapy failure, which included LIT + VEN (1), LIT + FLT3i (5), AZA + VEN + FLT3i (5), LIT (1), IC + FLT3i (3), cladribine regimen (1), finally all of them dramatically achieving CR and bridging to HSCT, further highlighting the pivotal role of FLT3i and VEN in salvage treatment of NUP98::NSD1 patients.

For NUP98::NSD1 patients, there was no significantly difference about 4-year OS rate between younger and older than 60 years (*p* = 0.239) (Fig. 2C). However, in non-NUP98::NSD1 patients, the 4-year OS rate of patients younger than 60 years was better than older than 60 years (*p* = 0.007) (Fig. 2D). In NUP98::NSD1 patients younger than 60 years old, there was no statistically difference about 4-year OS between IC alone group and IC + FLT3i/VEN group (*p* = 0.946), which was also the same as non-NU98::NSD1 patients (*p* = 0.094) (Fig. 2E, F). It should be mentioned that this result must be interpreted carefully due to the limitation of patient numbers. In addition, the 4-year OS rate of patients who received HSCT was significantly better than who did not receive HSCT, whatever in NUP98::NSD1 patients or non-NUP98::NSD1 patients (*p* < 0.001 and *p* = 0.002, respectively) (Fig. 2G, H).

In univariate analyses, AML patients received FLT3i and/or VEN therapy and HSCT showed a significant higher OS (*p* = 0.041 and *p* < 0.001, respectively), while FLT3, WT1, NUP98::NSD1 and disease status after initial induction therapy showed no significant OS disadvantage (*p* = 0.857, *p* = 0.941, *p* = 0.870 and *p* = 0.917, respectively) (Table S2). Multivariable Cox regression analyses for AML patients also showed that FLT3i and/or VEN therapy (HR = 0.013; 95% CI: 0.001~0.199; *p* = 0.002) and HSCT (HR = 0.003; 95% CI: 0.00006~0.099; *p* = 0.001) were significantly associated with improved OS. In addition, age (HR = 1.100; 95% CI: 10.32~1.171; *p* = 0.003), WBC (HR = 1.031; 95% CI: 1.008~1.054; *p* = 0.007) and FLT3 mutation (HR = 0.006; 95% CI: 0.0002~0.145; *p* < 0.001) were found to significantly associated with OS. NUP98::NSD1 (HR = 3.814; 95% CI: 0.333~43.737; *p* = 0.282) was similar to univariate analysis, maybe attributed to the salvage role of FLT3i and/or VEN introduction as well as HSCT in these patients treatment (Table S3).

Taken together, our study comprehensively demonstrated the clinical characteristics of a large cohort of NUP98r leukemia patients from 1491 AL patients for 4 years. In the total NUP98r AML patients, NUP98::NSD1 showed an initial poor response to standard chemotherapy but FLT3i and/or VEN introduction could save the depressed treatment outcome, and sequential HSCT could further improve the poor prognosis and balance the disadvantage of OS in this patients group.

### Supplementary information

Detail information of the 55 NUP98-rearranged patients including 51 AML and 4 ALL

Univariate analysis of clinicopathologic and molecular genetic features to predict overall survival

Multivariate analysis of 51 AML patients

Relationship between all mutations and NUP98 fusions in the 55 NUP98r leukemia patients.
