# Supplementary material for: The landscape of NUP98 rearrangements clinical characteristics and treatment response from 1491 acute leukemia patients
Source: Blood Cancer J. 2024 May 14;14(1):81. doi: 10.1038/s41408-024-01066-y (PMC11094082; doi:10.1038/s41408-024-01066-y)
Supplement: Supplementary file 1 — Detail information of the 55 NUP98-rearranged patients including 51 AML and 4 ALL [file 41408_2024_1066_MOESM1_ESM.pdf]

Table S1. Detail information of the 55 NUP98-rearranged patients including 51 AML and 4 ALL.

Case ID	Gender	Age, year	White blood cells (×109/L)	Hemoglobin (g/L)	Platelets (×109/L)	Blast cell in bone marrow (%)	FAB type	NUP98 partner	Karyotype	Other co-mutations	induction therapy	Disease status after induction therapy	Salvage Therapy	Optimal disease state	If received HSCT	Survival (day)
1	F	22	139.48	73	57	67	M5	NSD1	46,XX	FLT3-ITD p.L610_E611insVNEYFYVDFREYEYDLKWEFPRENL (VAF 3.7%) , MYC p.P75L (VAF 42.1%) , PTPN11 p.D61Y (VAF 41.2%)	IC	NR	/	NR	No	No (411)
2	F	19	369	94	79	76	M4	NSD1	36~45,XX,-7,-8,-15,-18,-20,-21,-22[cp12]/46,XX	FLT3-ITD p.N609_L610insWGEYDLKWEFPREN (VAF 27.3%)	IC	NR	LIT+FLT3i	CR	Yes	Yes (1320)
3	F	35	103.67	82	81	89.5	M4	NSD1	46,XX	FLT3-ITD p.N609_L610insVDFREYEYDLKWEFPREN (VAF 17.6%) , WT1 p.R375Gfs*6 (VAF 46.7%) , BCOR p.L984Q (VAF 45.7%)	IC	NR	LIT	CR	No	NA
4	F	40	58.26	92	96	72.5	M2	NSD1	46,XX	FLT3-ITD p.D600_L601insFREYEYD (VAF 27%) , MYC p.P74dup (VAF 30.2%) , WT1 p.V384Lfs*72 (VAF 9.3%)	IC	NR	LIT+FLT3i	CR	Yes	Yes (982)
5	M	42	25.97	80	79	78.5	M4	NSD1	46,XY,inv(9)c	FLT3-ITD p.E598_Y599insSNVDFREYE (VAF 6.8%) , FLT3-ITD p.E608_N609insYEYDLKWEFPRE (VAF 16.4%)	IC	NR	IC+FLT3i	CR	Yes	Yes (931)
6	M	26	76.92	95	35	83	M4	NSD1	32~45,XY,-6,-7,-15,-17,-18,-20,-21,-22[cp16]/46,XY	FLT3-ITD p.F612_G613insDNEYFYVDFREYEYDLKWEFPRENLEF (VAF 24.4%) , WT1 p.A387Gfs*69 (VAF 47.4%)	IC	PR	LIT+Ven	CR	Yes	Yes (931)
7	F	66	11.42	60	35	68.5	t-AML	NSD1	46,XX	FLT3-ITD p.F612_G613insGLVDFREYEYDLKWEFPRENLEF (VAF 15.7%) , DNMT3A p.H694P (VAF 43%) , WT1 p.R385Gfs*5 (VAF 31.8%) , ZBTB7A p.G582Qfs*64 (VAF 38.7%)	LIT+Ven	NR	LIT+Ven+FLT3i	CR	No	Yes (915)
8	F	65	168	66	65	46	AML	NSD1	46,XX	FLT3-ITD p.R595_E596insDGSSDNEYFYVDFR (VAF 33.9%)	IC	NR	LIT+FLT3i	CR	No	No (565)
9	M	52	113.7	127	102	74	M5	NSD1	46,XY	FLT3-ITD p.D600_L601insFREYEYD (VAF 41.3%)	IC	NR	LIT+Ven+FLT3i	CR	Yes	Yes (814)
10	M	39	82.19	75	47	74.5	M5	NSD1	46,XY	FLT3-ITD p.E598_Y599insTGSSDNEYFYVDFREYE (VAF 13.7%) , CEBPA p.A111Gfs*56 (VAF 21.3%) , WT1 p.S386* (VAF 7.6%)	IC	NR	/	NR	No	NA
11	F	61	4.66	70	62	29	M2	NSD1	46,XX	TET2 c.3594+1G>C (VAF 37.4%) , SMC1A p.R586Q (VAF 42.3%)	LIT+Ven	CR	/	CR	Yes	Yes (721)
12	F	55	3.95	98	41	86.5	M4	NSD1	46,XX	FLT3-ITD p.F594_D600dup (VAF 47.2%) , RUNX1 p.R166P (VAF 42.9%)	IC	PR	LIT+Ven+FLT3i	CR	Yes	Yes (709)
13	M	41	2.25	84	54	66.5	M4	NSD1	47,XY,+8/46,XY	WT1 p.S386* (VAF 7.4%)	IC	PR	LIT+Ven+FLT3i	CR	Yes	Yes (632)
14	M	26	142.5	107	48	63.5	M4	NSD1	46,XY	FLT3-ITD p.D593_F605dup (VAF 33.2%)	IC+FLT3i	PR	LIT+Ven+FLT3i	CR	Yes	Yes (543)
15	F	79	32.9	78	92	95	M4	NSD1	46,XX	FLT3-ITD p.N609_L610insPSMSTSRCREYEYDLKWEFPREN (VAF 35.3%) , ASXL1 p.R404* (VAF 44.9%) , TET2 p.Q943* (VAF 50.1%) , WT1 p.T382Afs*2 (VAF 7.3%) , WT1 p.L316Ffs*33 (VAF 54.5%) , RUNX1 p.W477L (VAF 10.6%)	LIT+Ven	CR	/	CR	No	No (285)
16	M	28	152.1	127	61	81.5	M5	NSD1	46,XY	FLT3-ITD p.Y597_E608dup (VAF 34.1%)	IC	NR	/	NR	No	No (63)
17	M	41	44.5	102	22	87	M5	NSD1	44~47,XY,+8,del(9q31)[cp10]	NRAS p.Q61R (VAF 26%) , FLT3-TKD p.D839G (VAF 6.9%) , WT1 p.S386* (VAF 3.4%) , NRAS p.G12D (VAF 3%)	IC	PR	LIT+FLT3i	CR	Yes	Yes (303)
18	F	36	184.96	63	50	55.5	M4	NSD1	45~46,XX,del(9)(q13q33),22ps+[cp4]/46,XX,22ps+[12]	FLT3-ITD p.F612_G613insADNEYFYVDFREYEYDLKWEFPRENLEF (VAF 80%) , WT1 p.A387Sfs*5 (VAF 51.8%) , WT1 p.Q197* (VAF 5.5%) , SETBP1 p.R1162Q (VAF 16.6%)	LIT+Ven	NR	/	NR	No	No (15)
19	F	65	200	60	80	90	M5	NSD1	46,XX	FLT3-ITD p.Y597_K614dup (VAF 29.6%) , TET2 p.P1013Dfs*22 (VAF 46.2%)	IC	NR	/	NR	No	No (11)
20	M	15	63.92	70	71	40	M5	NSD1	46,XX	FLT3-ITD p.P606_R607insGEYDLKWEFP (VAF 24.7%) , MYC p.P74_P75insRNP (VAF 21.9%)	IC	NR	IC+FLT3i	CR	Yes	Yes (238)
21	M	49	212	78	58	72	M4	NSD1	47,XY,+8[2]/46,XY[11]	FLT3-ITD p.R595_F605dup (VAF 39%)	IC	PR	IC+FLT3i	CR	No	No (81)
22	M	44	126	116	24	71	M4	NSD1	47,XY,+8[2]	FLT3-ITD p.D593_F594insYVTGSSDNEYFYVD (VAF 7%) , IDH1 p.R132H (VAF 13.6%)	IC	NR	/	NR	No	No (67)

Case ID	Gender	Age, year	White blood cells (×109/L)	Hemoglobin (g/L)	Platelets (×109/L)	Blast cell in bone marrow (%)	FAB type	NUP98 partner	Karyotype	Other co-mutations	induction therapy	Disease status after induction therapy	Salvage Therapy	Optimal disease state	If received HSCT	Survival (day)
23	M	36	95.55	120	167	69	M4	NSD1	46,XY	FLT3-ITD p.L610_E611insQMVQVTGSSDNEYFYVDFREYEYDLKWEFPRENL (VAF 37.9%) , FLT3-ITD p.E611_F612insPPQVTGSSDNEYFYVDFREYEYDLKWEFPRENLE (VAF 22.9%)	IC	NR	LIT+FLT3i	PR	Yes	Yes (989)
24	M	4	18.99	66	265	15.5	AML	NSD1	46,XY	CEBPA p.E59* (VAF 44.6%) , WT1 p.R467W (VAF 52.8%)	IC	NR	IC	CR	Yes	Yes (653)
25	M	8	234	44	37	79	M4	NSD1	46,XY	FLT3-ITD p.K614_V615insSSDNEYFYVDFREYEYDLKWEFPRENLEFGK (VAF 39.1%)	IC	NR	/	NR	No	Yes (349)
26	F	50	14.81	67	26	29	M2	HOXA9	46,XX,t(7:11)(p15;p15)/47,XX,t(7;11)(p15;p15),+M/46,XX	GATA2 p.N371_A372insPVCN (VAF 9.6%) , TET2 p.R1516* (VAF 4%) , MYC p.P74T (VAF 4%)	IC	NR	/	NR	No	No (13)
27	F	57	6.4	90	48	78	M2	HOXA9	46,XX,t(7;11)(p15;p15)/47,XX,t(7;11)(p15;p15),+8/46,XX	FLT3-TKD p.D835Y (VAF 23.1%) , GATA2 p.A372T (VAF 28%) , FAT1 p.E4573K (VAF 17.7%) , FAT1 p.S4207I (VAF 32.4%) , IL7R p.A254T (VAF 29.3%) , WT1 c.1355-2A>C (VAF 16.6%) , WT1 p.S386Lfs*71 (VAF 31.5%) , ZBTB7A p.A287V (VAF 19.4%) , RUNX1 p.D332N (VAF 29.1%) , NOTCH1 p.G1034S (VAF 17.7%)	IC	CR	/	CR	No	No (423)
28	M	24	43.13	97	83	46.5	M4	HOXA9	46,XY,t(7;11)(p15;p15)/46,XY	NRAS p.G13V (VAF 7.7%) , IDH1 p.R132H (VAF 46.4%)	IC	CR	/	CR	Yes	Yes (1096)
29	F	37	1.5	116	140	24	M2	HOXA9	46,XX,t(7;11)(p15;p15)/46,XX	TET2 p.Q1541* (VAF 23.2%) , TET2 p.E1958Rfs*57 (VAF 15.2%) , WT1 p.S386_E389delins* (VAF 21%)	LIT	CR	/	CR	Yes	Yes (1031)
30	F	28	47.66	28	20	28	AML	HOXA9	46,XY,t(7;11)(p15;p15)/46,XY	TET2 p.Y819* (VAF 87.6%)	IC	PR	IC	CR	Yes	Yes (924)
31	M	24	130.57	55	31	25.5	M2	HOXA9	46,XY,t(7;11)(p15;p15)	FLT3-ITD p.F621_G622insFREYEYDLKWEFPRENLEFGKNGMCQMFLQHFFSIGKSLKCTYSPFVFAGKVLGSGAF (VAF 21.4%)	IC	CR	/	CR	Yes	Yes (784)
32	F	52	74.14	87	31	14.5	t-AML	HOXA9	46,XX,t(7:11)(p15;p15)	KRAS p.G12V (VAF 44.1%) , BRCA1 p.P1099Lfs*10 (VAF 59.2%)	IC	CR	/	CR	Yes	Yes (725)
33	F	38	47.96	53	10	81	AML	HOXA9	46,XX,inv(3)(q21q26),t(7;11)(p15;p15),del(12p12),del(9p12)[cp11]	IDH2 p.R140W (VAF 49.2%)	LIT+Ven	CR	/	CR	Yes	Yes (261)
34	M	34	15.88	90	201	13.5	AML	HOXA9	NA	FLT3-ITD p.F612_G613insGFREYEYDLKWEFPRENLEF (VAF 5%)	IC	NR	LIT+Ven+FLT3i	CR	Yes	Yes (774)
35	F	66	2.55	87	103	18.5	AML	HOXA9	45~46,XX,t(7;11)p15;p15),21ps+[cp6]/46,XX,21ps+	IDH1 p.R132H (VAF 34.8%)	LIT+Ven	CR	/	CR	No	Yes (65)
36	M	30	151.55	68	33	13	AML	PRRX2	46,XY,t(9;11)(q34;p15)/46,XY	NRAS p.G12S (VAF 14.6%) , NRAS p.G12D (VAF 8.9%) , GATA2 p.A372T (VAF 41.3%) , KRAS p.G12D (VAF 15.9%)	IC	CR	/	CR	Yes	Yes (1015)
37	F	66	114.46	50	10	48.5	M4	PRRX2	46,XX	RUNX1 p.A251Dfs*11 (VAF 43.2%) , RUNX1 p.Q449Efs*152 (VAF 50.7%) , TET2 p.Q769Rfs*62 (VAF 29.3%) , NPM1 p.S260C (VAF 44%)	LIT+Ven	CR	/	CR	No	No (227)
38	F	59	18.43	84	21	32	AML	PRRX2	41~46,XX,inv(5)(q14q32)[cp6]/39~46,XX,inv(5)(q14q32),t(9;11)(q34;p15)[cp10]/44~46,XX,inv(5)(q14q32),der(7p12),-11,der(12)t(11;12)(q13;p13),-19,+M1~M2[cp3]	GATA2 p.A372T (VAF 32%) , WT1 p.R385Tfs*5 (VAF 47.6%) , RUNX1 p.S141_A142insG (VAF 49.1%) , NRAS p.G12D (VAF 45.4%)	LIT	NR	/	NR	No	No (19)
39	F	58	18.38	93	49	15.5	s-AML	PRRX2	45,XX,-7/45,XX,-7,t(9;11)(q34;p15)	WT1 p.A387Cfs*6 (VAF 34.5%) , ETV6 p.Y346Lfs*6 (VAF 38.1%) , PTPN11 p.F71L (VAF 10%) , PTPN11 p.S506P (VAF 19.8%)	LIT	CR	/	CR	Yes	No (283)
40	F	64	70.52	65	83	33	M4	HMGB3	46,XX	FLT3-ITD p.F612_G613insVKWEFPRENLEF (VAF 4.3%) , NRAS p.G12D (VAF 10.5%) , NRAS p.G13D (VAF 5.4%) , MYC p.TP73GL (VAF 13%) , KRAS p.G13D (VAF 6.4%) , IDH2 p.R140Q (VAF 5%)	IC	NR	/	NR	No	No (258)
41	F	45	92.45	73	56	88	AML	HMGB3	46,XX	FLT3-ITD p.Y599_D600insANVDFREYEY (VAF 50.8%) , WT1 p.Q257* (VAF 47.1%) , WT1 p.S386Vfs*9 (VAF 14.4%)	IC+FLT3i	CR	/	CR	Yes	Yes (518)
42	F	50	2.6	69	34	32	AML	TOP1	45~46,XX,t(11;20)(p15;q11)[cp10]	NRAS p.Q61R (VAF 47.6%) , STAG2 p.M1212* (VAF 47.6%)	LIT+Ven	CR	/	CR	No	Yes (375)

Case ID	Gender	Age, year	White blood cells (×109/L)	Hemoglobin (g/L)	Platelets (×109/L)	Blast cell in bone marrow (%)	FAB type	NUP98 partner	Karyotype	Other co-mutations	induction therapy	Disease status after induction therapy	Salvage Therapy	Optimal disease state	If received HSCT	Survival (day)
43	F	58	1.6	102	125	35.5	t-AML	TOP1	44~46,XX,del(7)(q31),t(11;20)(p15;q11)[cp8]/46,XX	FLT3-TKD p.D835V (VAF 6.4%) , CEBPA p.S299Efs*22 (VAF 5%) , NRAS p.G12D (VAF 5.1%)	IC	CR	/	CR	No	Yes (355)
44	M	48	5	91	42	21	s-AML	KDM5A	46,XY	FLT3-ITD p.T582_E604dup (VAF 12.6%) , CEBPA p.A72Rfs*89 (VAF 8.6%) , PTPN11 p.D61H (VAF 16%) , ASXL2 p.E416* (VAF 11.2%) , WT1 p.H416Y (VAF 15.5%) , IDH1 p.R132H (VAF 5%)	IC	CR	/	CR	Yes	Yes (364)
45	F	26	79.13	96	84	74	M5	KDM5A	46,XX	RAD21 p.G547Afs*65 (VAF 21.6%) , FLT3 p.V491L (VAF 10.5%) , FLT3 p.K663R (VAF 9.3%)	IC	CR	/	CR	Yes	Yes (284)
46	M	28	6.29	55	250	57	ETP-ALL	CCDC28A	47~48,XY,+4,del(6q22)[cp5]/46,XY	NRAS p.G12D (VAF 49.6%) , KIT p.D496Y (VAF 33%) , WT1 p.R218Tfs*35 (VAF 38.2%) , ETV6 p.W380L (VAF 46.7%) , SUZ12 c.1024-1G>T (VAF 44.9%) , PHF6 p.R274* (VAF 44.2%)	VDPCP	CR	/	CR	No	No (72)
47	M	21	3.97	136	127	32	ETP-ALL	CCDC28A	47,XY,del(6q21),+8/46,XY	CEBPA p.H24Afs*84 (VAF 24.5%) , PHF6 p.E177Qfs*42 (VAF 24%) , RUNX1 p.D332N (VAF 25.3%)	IA	CR	/	CR	Yes	Yes (289)
48	M	54	7.23	89	92	79.5	M4	TNRC18	47,XY,+8/46,XY	NRAS p.Q61H (VAF 21.7%) , IDH1 p.R132C (VAF 42.4%) , SMC1A p.R586Q (VAF 41.4%)	IC	NA	/	NA	No	NA
49	F	32	89.79	74	15	52	M4	HOXA11	NA	PTPN11 p.L261F (VAF 40.3%)	IC	NR	/	NR	No	No (23)
50	M	32	83.79	29	5	27	M2	HHEX	44~46,XY,del(7)(q31),t(10;11)(q23;p15),-18,+M1~M2[cp11]	FLT3-ITD p.V581_E611dup (VAF 15.6%) , FLT3-ITD p.P606_R607insSPVDFREYEYDLKWEFP (VAF 12.9%) , FLT3-ITD p.D593_R607dup (VAF 10%)	IC	NA	/	NA	No	Yes (464)
51	M	70	14.88	123	136	21	s-AML	DDX10	43~46,XY,inv(11)(p15q22)[CP8]/46,XY	CEBPA p.M1? (VAF 11.9%) , CEBPA p.E8* (VAF 18.7%) , CEBPA p.H24Afs*84 (VAF 11.7%) , CEBPA p.I55Afs*54 (VAF 8.9%) , CEBPA p.Q207* (VAF 11%)	IC	CR	/	CR	No	NA
52	F	74	2.79	43	105	65	M4	PSIP1	46,XX	No other mutation	LIT	NR	/	NR	No	No (16)
53	M	34	4.18	85	213	16	M2	KMT2A	42~46,XY,del(11q23)[cp13]	DNMT3A p.R882H (VAF 42.9%) , IDH2 p.R140Q (VAF 2.6%)	IC+Ven	CR	/	CR	Yes	Yes (221)
54	M	36	3.64	64	105	93	B-ALL	ASNSP4	46,XY	IDH1 p.R132C (VAF 48.7%)	VDPCP	NR	/	NR	No	No (95)
55	M	13	244.67	86	267	94	ETP-ALL	LNP1	45~47,XY,t(3;11)(q12;p15),+8[cp13]/46,XY	FLT3-TKD p.D835H (VAF 6.3%) , FLT3 p.Y572C (VAF 41.9%) , ETV6 p.I140Gfs*71 (VAF 50%) , CBL p.Y774* (VAF 11.7%)	CDVD+Asp+CAG	NR	/	NR	No	No (80)

Abbreviations: F, female; M, male, FAB, French-American-British; AML,acute myeloid leukemia; t-AML,therapy-related AML; s-AML, secondary AML; ALL, acute lymphoblastic leukemia; ETP-ALL, Early T-cell precursor acute lymphoblastic leukemia; B-ALL, B-cell acute lymphoblastic leukemia; Ven, venetoclax; FLT3i, FLT3 inhibitor; VDPCP; vincristine,daunorubicin, asparaginase (ASP), cyclophosphamide and prednisone; CDVD, cyclophosphamide, daunorubicin, vincristine and dexamethasone; HSCT, hematopoietic stem cell transplantantion, CR, complete remission; PR, partial remission; NR, no remission; NA, not available. IC, Intensive Chemotherapy, including idarubicin+Ara-C±Etoposide, homoharringtonine+Ara-C±etoposide, daunorubicin+Ara-C±etoposide, Mitoxantrone+Ara-C, cladribine and homoharringtonine+low dose Ara-C+aclacinomycin+G-CSF; LIT, Low Intensive Therapy, including azacitidine, decitabine, hydroxyurea, low dose Ara-C+idarubicin+G-CSF.
