# Supplementary material for: The landscape of NUP98 rearrangements clinical characteristics and treatment response from 1491 acute leukemia patients
Source: Blood Cancer J. 2024 May 14;14(1):81. doi: 10.1038/s41408-024-01066-y (PMC11094082; doi:10.1038/s41408-024-01066-y)
Supplement: Supplementary file 2 — Univariate analysis of clinicopathologic and molecular genetic features to predict overall survival [file 41408_2024_1066_MOESM2_ESM.pdf]

Table S2.Univariate analysis of clinicopathologic and molecular genetic features to predict overall survival.

Variables	No.of patients, n(%)	*p* value
Age		0.103
⩾60 years	10 (18.2)	
<60 years	45 (81.8)	
Gender		0.088
Male	27 (49.1)	
Female	28 (50.9)	
White blood cells		0.570
Leukocytosis	40 (72.7)	
Leukepenia	9 (16.4)	
Normal	6 (10.9)	
Hemoglobin		1.000
Anemia	50 (90.9)	
Normal	5 (9.1)	
Platelets		0.820
Thrombocytopenia	41 (74.5)	
Normal	14 (25.5)	
AL type		
AML	51 (92.7)	
ETP-ALL	3 (5.5)	
B-ALL	1 (1.8)	
Karyotype		0.861
Complex(⩾3 aberrations)	3 (5.7)	
Simple (1~2 aberrations)	26 (49.1)	
Normal	24 (45.3)	
Gene mutations*		
FLT3	32 (58.2)	0.917
FLT3-ITD	27 (49.1)	0.856
WT1	17 (30.9)	0.794
NUP98 partner		0.263
NSD1	25 (45.5)	
HOXA9	10 (18.2)	
Other	20 (36.4)	
FLT3 inhibitor**		0.072
Yes	17 (33.3)	
No	34 (66.7)	
Ven**		0.192
Yes	19 (37.3)	
No	32 (62.7)	
FLT3i or/and Ven therapy**		0.041
Yes	26 (51)	
No	25 (49)	
Status of initial induction therapy**		0.917
CR	19 (37.3)	
PR/NR	30 (58.8)	
NA	2 (3.9)	
HSCT**		<0.001
Yes	26 (51)	
No	25 (49)	

Abbreviations: AL, acute leukemia; AML,acute myeloid leukemia; ETP-ALL, Early T-cell precursor acute lymphoblastic leukemia; B-ALL, B-cell acute lymphoblastic leukemia; FLT3, Fms-like tyrosine kinase 3; FLT3-ITD, internal tandem duplications of the FLT3 gene; WT1, Wilms tumor 1; FLT3i, FLT3 inhibitor; VEN, venetoclax; CR, complete remission; PR, partial remission; NR, no remission; NA, not available; HSCT, hematopoietic stem cell transplantation. *, Only listed high frequency mutations. **, Only analyzed in 51 AML patients.
