# Supplementary material for: The landscape of NUP98 rearrangements clinical characteristics and treatment response from 1491 acute leukemia patients
Source: Blood Cancer J. 2024 May 14;14(1):81. doi: 10.1038/s41408-024-01066-y (PMC11094082; doi:10.1038/s41408-024-01066-y)
Supplement: Supplementary file 3 — Multivariate analysis of 51 AML patients [file 41408_2024_1066_MOESM3_ESM.pdf]

Table S3. Multivariate analysis of 51 AML patients.

Cox regression analysis - Overall survival				
Parameters	Hazard ratio	95% confidence interval		*p* value
		Lower limit	Upper limit	
Gender	0.543	0.065	4.511	0.572
Age(y)	1.100	1.032	1.171	0.003
White blood cell (×109/L)	1.031	1.008	1.054	0.007
Hemoglobin (g/L)	1.031	0.993	1.072	0.114
Platelet (×109/L)	1.009	0.984	1.035	0.488
Bone marrow blast cell (%)	1.017	0.981	1.055	0.355
WT1 mutation	4.207	0.613	28.877	0.144
FLT3 mutation	0.006	<0.001	0.145	0.002
NUP98::NSD1 fusion gene	3.814	0.333	43.737	0.282
FLT3i or/and VEN therapy	0.013	0.001	0.199	0.002
HSCT	0.003	<0.001	0.099	0.001

Overall survival: time from confirmed diagnosis to last follow-up or death. Abbreviations: *WT1, Wilms tumor 1; FLT3, Fms-like tyrosine kinase 3* ; HSCT, hematopoietic stem cell transplantation.
