# Supplementary material for: The landscape of NUP98 rearrangements clinical characteristics and treatment response from 1491 acute leukemia patients
Source: Blood Cancer J. 2024 May 14;14(1):81. doi: 10.1038/s41408-024-01066-y (PMC11094082; doi:10.1038/s41408-024-01066-y)
Supplement: Supplementary file 4 — Relationship between all mutations and NUP98 fusions in the 55 NUP98r leukemia patients. [file 41408_2024_1066_MOESM4_ESM.pptx]

## Slide 1
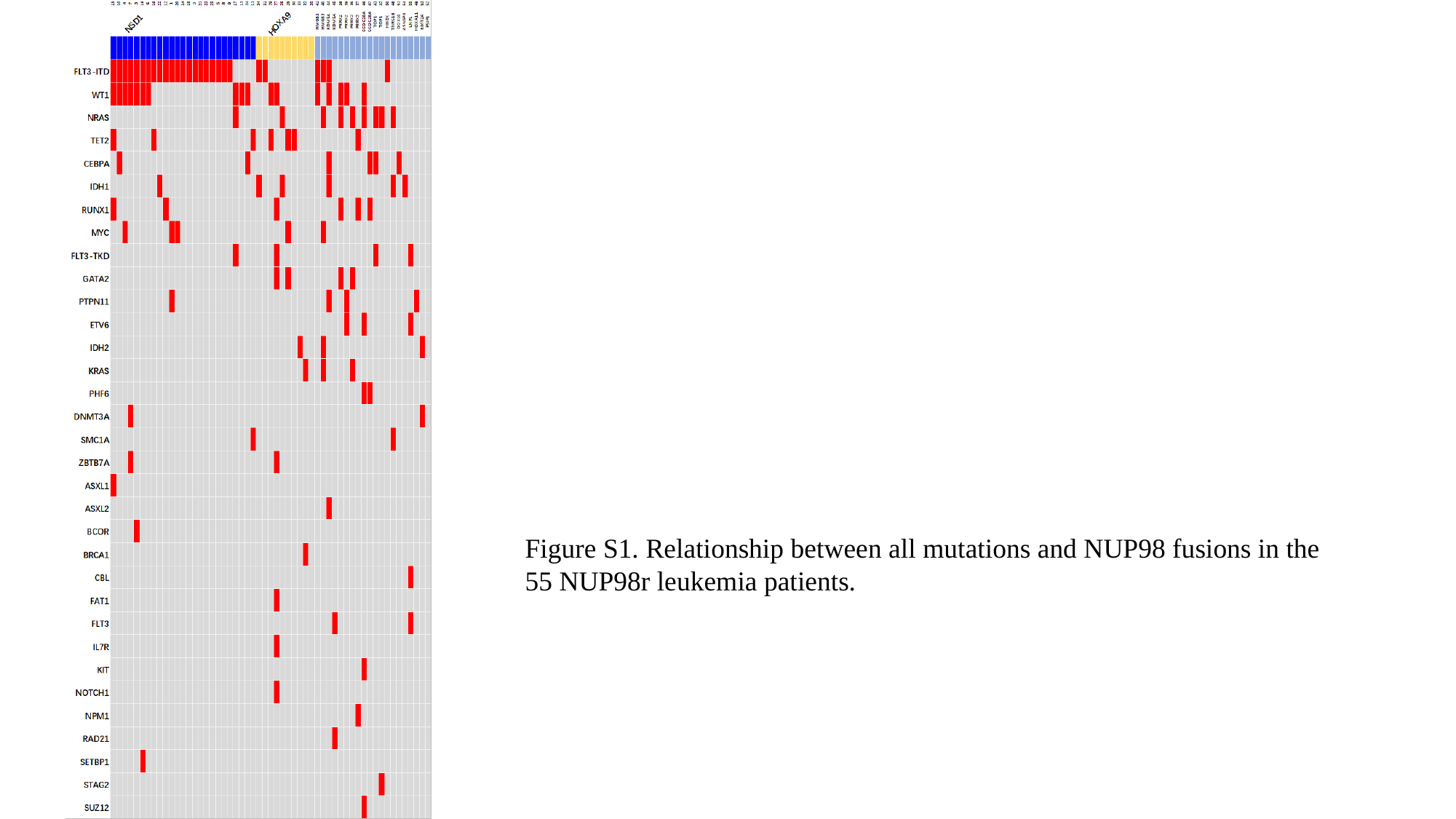

Figure S1. Relationship between all mutations and NUP98 fusions in the 55 NUP98r leukemia patients.

## Slide 2
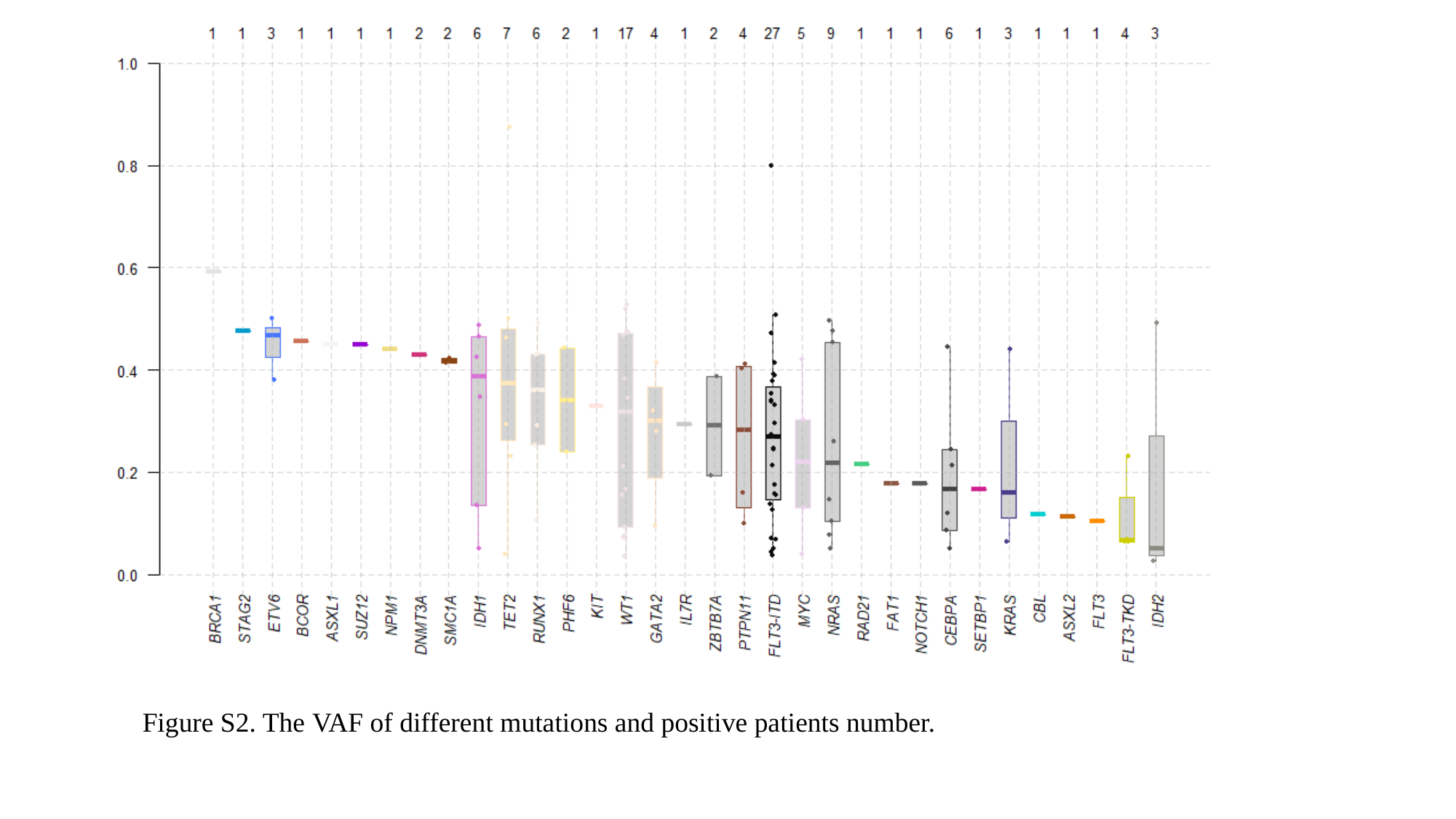

Figure S2. The VAF of different mutations and positive patients number.
